# “Query.”—What We Want to Know

**Published:** 1896-08

**Authors:** 


### "QUERY."—WHAT WE WANT TO KNOW.

JUNE number of the JOURNAL, page 508.

How four prominent veterinarians failed in an examination for soundness to note the existence of scirrhous cord in the trotting-bred horse "Sheraldine," recently sold at auction at Chicago?

---

EDITOR JOURNAL OF COMPARATIVE MEDICINE, ETC.

UNDER the heading "What we Want to Know," page 508 of JOURNAL for June, how four prominent veterinarians failed to note the existence of scirrhous cord in the trotting-bred horse "Sheraldine," recently sold at auction at Chicago.

Permit me to inform the editors that the prominent veterinarians referred to in this case did not fail to note the existence of scirrhous cord, as the horse had been examined by Dr. Hughes for a purchaser and rejected by him on account of the existence of scirrhous cord. Could any conscientious veterinarian do other than report to buyer, on detecting the same conditions existing upon examination, that said animal was certainly unsound? The buyer declining to take possession of the horse, the seller or party conducting the auction being annoyed at the decision of Dr. Hughes, called in a prominent veterinarian to examine the horse, as he was not satisfied that Dr. Hughes should have proclaimed the horse unsound under the existing conditions. Imagine the smile on the face of the seller when the prominent veterinarians disagreed with Dr. Hughes and declared that the horse with the existing scirrhous cord was sound.

It is nevertheless true that prominent veterinarians can be found in Chicago and elsewhere who at times will differ in opinion regarding an unsound horse, but, this being a decision so contrary to common usage that it can be no surprise to your readers when you come out so plainly in your columns and seek for information regarding the case referred to, considering that such an absurd opinion had been given that a horse with an existing scirrhous cord had been proclaimed sound by a prominent veterinarian of Chicago.

Instead of being so indefinite in your remarks regarding four prominent veterinarians of Chicago being unable to detect scirrhous cord in examination of a horse for soundness, I think it would be only justice to the veterinarians of Chicago for the editors of the JOURNAL to make known the names of those whose actions cast reflection upon the veterinarians of our city, and in the future it would be well for the editors to be more particular and not multiply 1 x 4, making us four times worse than we are.

Yours respectfully,

ROBERT G. WALKER, M.D.C.

CHICAGO, ILL., July 10, 1896.

---

EDITOR JOURNAL OF COMPARATIVE MEDICINE, ETC.

I BEG to take exception to an article published in the June number of the JOURNAL under the heading "What We Want to Know." The second paragraph sets forth the accusation that four prominent veterinarians of Chicago failed to discover scirrhous cord in an examination for soundness of the trotting-horse "Sheraldine." How such an article ever reached the JOURNAL is a mystery to those acquainted with the circumstances of this case, which, owing to the amount of money involved, caused no little discussion among the veterinarians and horsemen of this city. The facts are so different from the allegations of this short but spicy article, as the first veterinarian who examined "Sheraldine" after his sale discovered what he considered a scirrhous cord. Other veterinarians who were consulted later pronounced the lesion a natural sequence of recent castration, and their opinion has since proved to be correct. Did the writer wish to reflect upon the Chicago veterinarians as a whole, or only on those whose opinion proved so valuable to both the buyer and seller of this horse, or was the injury intended for the present owner of "Sheraldine?"

Very truly yours,

L. A. MERILLAT.

CHICAGO, ILL., June 29, 1896.

On to Buffalo!

